# Regional differences in the biological variability of plantar pressure as a basis for refining diagnostic gait analysis

**DOI:** 10.1038/s41598-024-53787-6

**Published:** 2024-03-11

**Authors:** Ewa Latour, Emilia E. Latour, Jarosław Arlet

**Affiliations:** Department of Physiotherapy, Poznan University of Physical Education, 61-871 Poznań, Poland

**Keywords:** Biophysics, Medical research

## Abstract

The variability of movement plays a crucial role in shaping individual's gait pattern and could, therefore, potentially serve diagnostic purposes. Nevertheless, existing concepts for the use of variability in diagnosing gait present a challenge due to the lack of adequate benchmarks and methods for comparison. We assessed the individuality of contribution of foot parts that directly mediate the transmission of forces between the foot and the ground in body weight shifting during walking based on 200 pedobarometric measurements corresponding to the analysed foot parts for each of 19 individuals in a homogeneous study group. Our results show a degree of individualisation of the contribution of particular foot parts in the weight-shift high enough to justify the need to consider it in the diagnostic analysis. Furthermore they reveal noticeable, functionally driven differences between plantar areas most apparent between the lowest individuality for the first foot ray and the highest for second one and metatarsus. The diagnostic reference standard in pedobarometry should describe the contribution in the shift of body weight during walking for each area of the foot separately and include information on the intra-individual variation and individualisation of descriptors of the contribution. Such a comprehensive standard has the potential to increase the diagnostic value of pedobarometry through enrichment of the assessment description.

## Introduction

Walking is one of the most accurately repetitive periodic movement tasks. Its high repeatability comes from the individual optimisation of this task in terms of energy and efficiency, when adjusting to counter environmental influences^1,2^. The consistency of these repeated movements is a marker for the quality of control an individual has over their walking^3^, which can be indicative of the health of that individual. Evaluating this consistency, based on the comparison of the temporal, spatial, and kinetic variability of the gait descriptors of individuals against an identified physiological standard, offers the potential for the development of effective diagnostic tools^4,5^. While measuring these descriptors is not hampered by any technical problems, the theoretical framework for interpreting the measurement results for diagnostic purposes is limited because of discrepancy in the outcomes of studies comprising manifestations of various gait characteristics in results obtained from measurements depicting the process of body weight shifting^6,7^.

To be effectively used as diagnostic tools, measurement data must be accompanied by a parametrised description that allows for the comparison of different individuals, as well as for the establishment of criteria that can assess the deviation of descriptor values related to a given individual from a population benchmark. For such standard criteria to be effective, it is imperative that their creation is informed by biological variation^8^. This entails the calculation of both intra- and inter-individual variability. It is important to note that both these types of variability are mediated by common factors, genetic and environmental, contributing to the generalised views that “intra- and inter-individual variability are inextricably bound together”^9^. With this in mind, determining the ratio between the contribution of intra- and inter-individual variability can be used to calculate the so-called index of individuality^10^. This index allows for an estimation of the expected degree of variation of the descriptors of bodily functions in individuals, the criteria for diagnostic assessment of the measured parameters, and determines how the criteria should be calculated—the greater the individuality of the descriptors, the more the calculation of these criteria should rely on individual-based variation and less on population-based variation, and vice versa^9–11^. Probability calculation provided characteristic values of this index for determining how to proceed with a diagnostic assessment. Only for individuality index above 1.4 population-based reference interval are appropriate for detecting pathologic changes for index value between 0.6 and 1.4 it should be use with caution and under 0.6 subject-based reference interval, i.e. estimated from examined person. Awareness of the need to account for biological variation in diagnostic analysis usually comes from researchers working in biochemical diagnostics^12^. Such an approach has been applied much less frequently to neurological^13^, psychological^14^, neurophysiological^15^ or biomechanical diagnostics^1,16^, although some studies encourage its adoption^8,17^.

The individuality of movement is subject to motor control theories emphasising the functional role of motion variability^18–20^ in shaping idiosyncratic, adaptive patterns of motor tasks^20,21^. Motor tasks are shaped through the revision of motor commands through error-based reduction of redundancy in the motor command space^22^. Through this process, the number of degrees of freedom (DOF’s) of a movement decreases, consequently decreasing the task-relevant variability as the process approaches its “endpoint”, which theoretically maximises the effectiveness and efficiency of the task^23^. It remains unclear how overall variability is contributed to by task-irrelevant variability of movements that do not affect the desired goal^19^, although some researchers view task-irrelevant variability level as a higher contributor than task-relevant variability^24^. This difference in contributions may be an effect of individual variation of the contribution of specific body segments to a particular motor task. Taking into account the location of a particular segment in the body structure, the DOF of each segment presumably reduces to a different extent in different individuals following personally unique processes of performance adjustment^25–27^. Thus, when considering movement task performance in the general population, one has to expect segment-specific values of the mean, intra-individual and inter-individual variation of the movement descriptors. Furthermore, the ratio of these two types of variation may vary from one body segment to another. This individuality of segmental functioning necessitates the tailored creation mode of diagnostic assessment criteria for each segment.

The manner of human walking manifests in the spatial and temporal distribution of forces on the plantar surface of the foot. This process is often analysed through measurement data obtained with pedobarometry. The high informativeness of such data has allowed researchers to establish that there is a high level of idiosyncrasy in anatomical and functional features of the foot, manifested in the processes of both standing and of body weight shifting^28,29^. Step-by-step analysis of the variation within plantar pressures found that the minimum number of measurements required for reliable analysis results is 200^30^. There is a generally positive correlation between dispersion and mean plantar pressure from successive measurements, the ratio of which can be expressed using a coefficient of variation (CV), which is higher in areas with lower mean plantar pressure values, such as around the periphery of the foot and at the midfoot, but lower in the heel and forefoot^31^. Detailed descriptions of the measurement data for precisely identified anatomical-functional areas of the foot should enable the characterisation of the inner functioning of the foot in walking and analysis of variation should enable to establish appropriate criteria of diagnostic assessment. However no concept for the use of these possibilities to provide diagnostically structured analytic methods has yet emerged. This is likely a factor behind the widespread belief that pedobarometry has little diagnostic value^32^. The inconsistency of conclusions from previous studies might be caused by the limited research on physiological gait that encompass biological variation linked to the functional differentiation of individual foot parts, including the individualisation of their functioning concerning their roles. Overcoming this challenge will require the replacement of the current diagnostic benchmark description, defined in terms of means and accompanied dispersions, with a standard which includes individuality indices for different foot parts. Determining the value this would bring to pedobarometry diagnostics requires calculating whether the value of these indices exceeds the minimum that would justify such a course of conduct.

Taking the above observations and assumptions together, it seems that the differentiation of the roles of various foot parts should manifest itself in the results of the pedobarometric measurements. This is due to different foot parts undergoing unique processes of adaptive reduction of their working variability. The extent of such processes is determined by the functional needs^33^ of each part within the cooperative process of body weight shift that takes place within the overarching parent structure of the foot. Therefore, we hypothesised that the index of individuality for pedobarometric descriptors of the work of different foot parts in shifting the body weight during walking should differ between them and fall within some characteristic range of values. To verify this hypothesis, we undertook to characterise the stepwise variability of plantar pressures on parts of the foot representing the bones interacting with the ground during comfortable walking under conditions that minimise the component of variability caused by factors other than the differentiation of the movement pattern shaped by motor learning. Following this assumption, we conducted a study on a carefully selected group of young adults, highly homogeneous in terms of age, gender, cultural environment and lifestyle, who had already developed a mature gait pattern but had not yet shown any signs of degeneration.

## Methods

For different anatomical-functional areas of the foot, measurements were made of the interactions of their plantar surface with the ground during body weight shift in free gait. This information was used to create an index describing the involvement of each foot part in body weight shifting, and the specific variation around the mean value for each foot area.

### Data acquisition

#### Participants

We examined nineteen young women of one nation, students of the same year of physical education university, who are physically active but not involved in sport, and had no observable signs of disease that could affect the musculoskeletal function. This group was characterized by the following values of age, mass, height, and BMI, respectively: 19.7 (SD = 0,6) years, 60.33 (SD = 6.81) kg, 1.67 (SD = 0.04) m, 21.58 (SD = 2.44) kg/m^2^. All had a similar lifestyle, being physically active but not involved in sport. Each participant gave informed consent prior to their participation in the study. The study was performed in accordance with the guidelines of the Declaration of Helsinki. The measurement protocol used in this study was approved by the institutional review boards of the Ethics Committee of the Poznan University of Medical Sciences (Act 1068/16 Archived Number 10/November/2016).

#### Equipment

**Figure 1:**
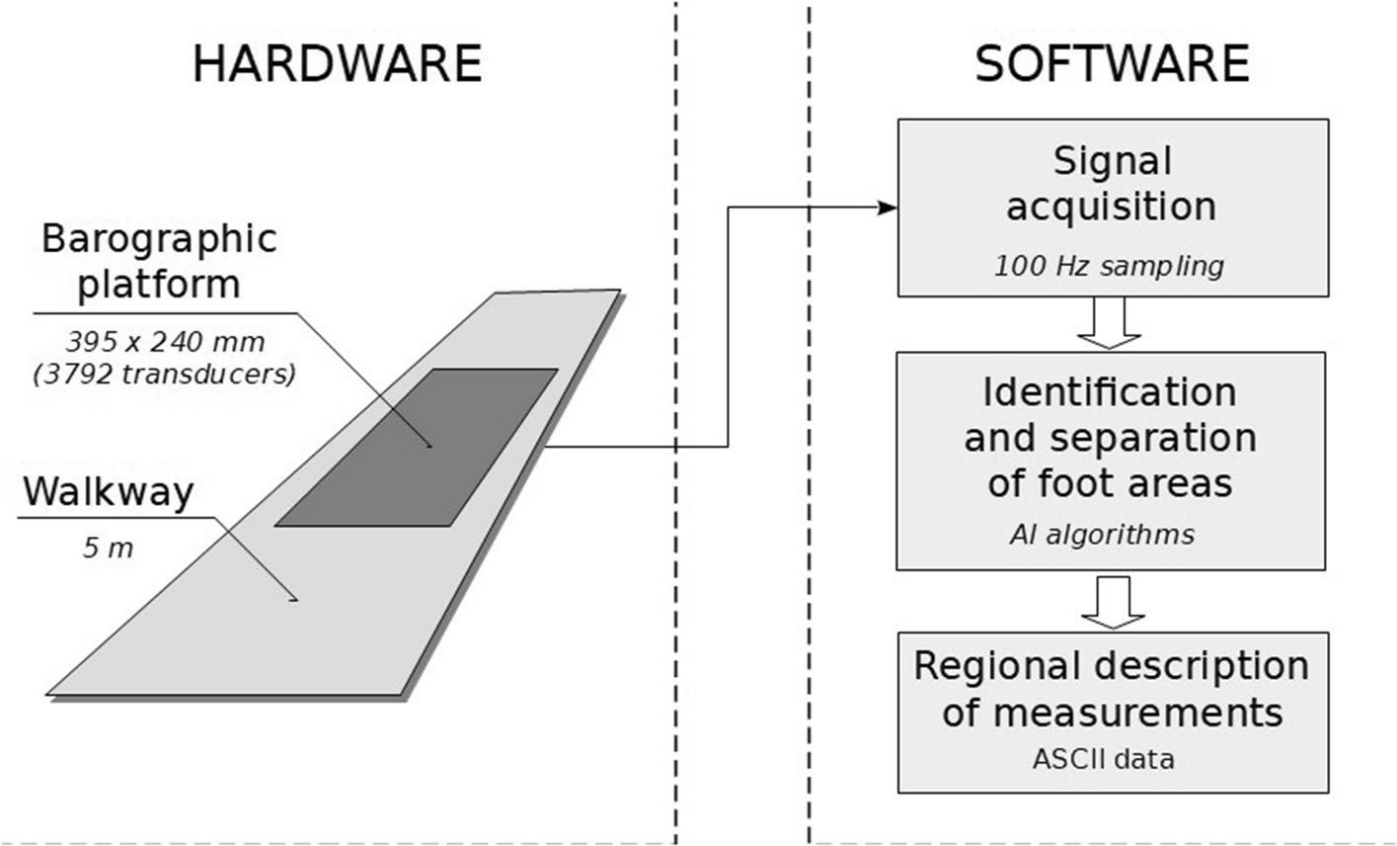
Scheme of the measurement system.

To measure changes in plantar pressure the following measurement tools were used (Fig. 1).

- emed-m pedobarometric platform, Novel, Munich, Germany (active area: 395 × 240 mm; 3792 capacitance-based force transducers at a resolution of 4 sensors/cm^2^), embedded in 5-m walkway,
- emed-m Novel software that collects measurement data with a sampling frequency of 100 Hz and performs a preliminary analysis of the obtained temporal-spatial distribution of foot plantar pressures.

#### Measurement protocol

Measurement data was obtained using the two-step method, in which the distribution of plantar pressures is registered during the stance phase of the second step^34^. During familiarization with the testing procedure participants were instructed to look straight ahead to avoid targeted movement that might alter the gait. Following current recommendations^30^ regarding the number of repetitions needed, we recorded 200 steps of each participant—100 each for the left and right foot, taken alternately.

#### Data extraction

The emed-m software calculated a set of indices describing the process of body weight shifting through the plantar surface of the foot for ten plantar areas (masks) separated with the use of Novel proprietary artifical inteligence algorithms, labelled with numbers: 1—heel (H), 2—midfoot (MF), 3–7—the five metatarsal heads (MTH1–MTH5), 8—hallux (first toe), 9—second toe, 10—third to fifth toes, and 0—for the entire foot surface. Using the AWK programming language, we extracted the values of maximal area (*mxa*) [cm^2^] and mean force (*mef*) [N] from the textual descriptions of the measurement results. Additionally, we extracted the contact time of the stance phase to assess the speed efect.

### Statistical analysis

We performed and developed our analyses and charts with the R Language and Environment for Statistical Computing^35^.

Before the measurement data was processed for statistical description and other analyses, we performed a qualitative assessment of the initial results. A Kendall’s rank correlation test of the measured values with the number of repetition in the 380 measurement sessions (19 participants × 2 feet × 10 masks) of sample size 100 did not show a noticeable learning effect (Kendall’s τ = − 0.025 [− 0.004 − 0.001], p-value = 0,33 [0.29 0.38]). A Kendall’s rank correlation test of stance phase time with other measured quantities showed no influence from gait velocity on the values of the measured parameters. A Shapiro–Wilk’s test of distribution normality did not show that the measurement data shows strong non-conformities with a normal distribution.

In order to properly compare the parameter values of different individuals, we normalised the data derived from the measurement system and calculated the percentage mean pressure (pmp) index. This is defined as the percentage ratio between the mean pressure values for specific masks and the total value for all registered areas of plantar pressures, according to the formula:

1 $${pmp}_{n}=100\cdot \frac{\frac{{mef}_{n}}{{mxa}_{n}}}{\frac{{mef}_{0}}{{mxa}_{0}}}=100\cdot \frac{{mef}_{n}\cdot {mxa}_{0}}{{mef}_{0}\cdot {mxa}_{n}}$$

where: *mef*_n_ is the mean force for mask *n*, *mef*_0_ is the mean force for the entire foot, mxa_n_ is the maximal area for mask n, mxa_0_ is the maximal area for the entire foot.

This index represents the contribution of foot parts to body weight shifting in each step.

To avoid the effects of outliers that might occasionally occur due to non-standard events, which the researcher missed while conducting the measurement, we subjected all datasets to adaptive winsorisation. This procedure defines outliers as values outside the interval defined by the lower limit of the difference between the first quartile and the 1.5 interquartile range and the upper limit of the sum of the third quartile and the 1.5 interquartile range. The winsorisation process converts these outliers to the extreme values of the lower and upper limit of the interval, which they lie beyond.

To select a suitable variability measure for a trustworthy comparison of several measurement datasets, we initially assessed the potential effect of mean values on their variability by using the Kendall correlation test. Since the positive correlation coefficient value indicated a strong effect, we implemented the coefficient of variation (CV), enabling the independent of the mean evaluation of variability as a variability measure in statistical analysis. This measure normalises variability with the mean value, according to the formula:

2 $$CV\left(x\right)=\frac{sd\left(x\right)}{mean\left(x\right)},$$

where: *x *is the set of values, *sd(x)* is the standard deviation of *x*, *mean(x)* is the mean of *x.*

For each mask of each participant’s foot, we estimated:

3 $${\mathrm{individual \,mean \,}{\text{IM}}}_{i,j,m}=mean({pmp}_{i,j,m})$$

and

4 $${\mathrm{individual \,CV \,}ICV}_{i,j,m}=CV({pmp}_{i,j,m})$$

followed by indices characterising each mask for the entire group of participants: the intra-individual mean (${A}_{m}$), representing the group-wide average values for each mask, the intra-individual variation (${V}_{m}^{intra}$) representing the mean CV for "trial-by-trial" values of a given participant’s index, and the inter-individual variation (${V}_{m}^{inter}$) representing the CV for the means of these values from all participants, according to the formulae:

5 $${{\text{intra}}-\mathrm{individual \,mean \,}A}_{m}=mean{({\text{IM}}_{i,j})}_{m}$$

6 $${{\text{intra}}-\mathrm{individual \,variation \,}V}_{m}^{intra}={mean({ICV}_{i,j})}_{m}$$

7 $${{\text{inter}}-\mathrm{individual \,variation \,}V}_{m}^{inter}=CV{({\text{IM}}_{i,j})}_{m}$$

where: *i* is the number assigned to a particular participant, *j* is the foot (left or right), *m* is the mask.

To characterise the diversity in the distributions of the values of the pmp index across the masks, we juxtaposed A with V^inter^, taking into account the standard deviations of the sets of means (IM) and CVs (ICV) of each participant’s measurement series. It allows us to use ANOVA analysis to estimate the effect size (η^2^) of the differences in the mean values across participants between masks and across masks between the participants.

Based on previous studies^8^, we estimated the biological variation in the analysed index for each area of the foot plantar surface. The authors of these works described the level of the variation by a quotient of intra-individual to inter-individual CV (V^intra^ to V^inter^), calling it the “index of individuality”. They also recommended that the characteristic values of this index be used as decision criteria when determining how to proceed with a diagnostic assessment. Contrary to its name, the index thus defined takes on higher values with data sets that differ less from each other and lower with those that differ more. Without departing from the ideas presented in previous works, for a better, intuitive understanding of the concepts presented in this paper, we took as an index of individuality the natural logarithm of the inverse quotient: inter-individual to intra-individual CV. This provided a more straight interpretation of higher values of the index of individuality as describing more individualised data and lower values as less individualised. Logarithmic rescaling simplifies the interpretation of the index of individuality as the fractional predominance of V^inter^ over V^intra.^ Thus rescaled index is symmetric around a value of 0 denoting equality between the V^intra^ and V^inter^. Positive values indicate the dominance of V^inter^ over V^intra.^, whereas negative values demonstrate the opposite relation. Our index of individuality (IxI) for mask *m* is calculated as follows:

8 $${IxI}_{m}={\text{ln}}(\frac{{V}_{m}^{inter}}{{V}_{m}^{intra}})$$

Accordingly, the formerly applied reference ranges for comparative procedures in the diagnostic evaluation take on a new description. The range below the decision criterion value of 0.6, considered a strong rationale for making assessments with respect to a subject-based (estimated from an examined person) variability transforms to the range above ln(1/0.6) = 0.512. Analogically the range above 1.4 is considered a strong rationale for making assessments based on population variability transforms to the range below ln(1/1.4) =  − 0.336.

Treating intra-individual variability as the second main measurement outcome description index alongside the mean, we also calculated the second-order inter-individual variability index for each mask, as the CV of CVs in each participant, according to the formula:

9 $${V}_{m}^{interII}=CV{({\text{ICV}}_{i,j})}_{m}$$

and further, the index of variation individuality (IvI), as follows:

10 $${IvI}_{m}={\text{ln}}(\frac{{V}_{m}^{interII}}{{V}_{m}^{intra}})$$

In order to gain an insight into the proposed functional differentiation of each part of the foot during weight transfer during gait, as outlined in the hypothesis, we investigated the individuality (IxI) of the pmp index in relation to its intra-individual variability (V^intra^). To illustrate the distribution of IxI values in relation to V^intra^ values across each anatomical and functional area of the foot, we standardized the value sets for both indices according to the formula:$std(x)=\frac{x-\overline{x} }{sd(x-\overline{x })}$. We then applied the k-means clustering method using the Hartigan-Wong algorithm^36^ to classify their associations. We presented the results of this classification on a chart and a map of the anatomical-functional areas of the foot.

To evaluate the influence of differences in biological variability of foot function between particular foot regions on diagnostic assessment, we presented the values of IxI and IvI indices with above mentioned -0,336 and 0,512 decision criteria.

We described all data sets analyzed, providing their means and their 95% confidence intervals (CIs), the borders of which we present in square brackets ([CIlower CIupper]).

## Results

**Figure 2:**
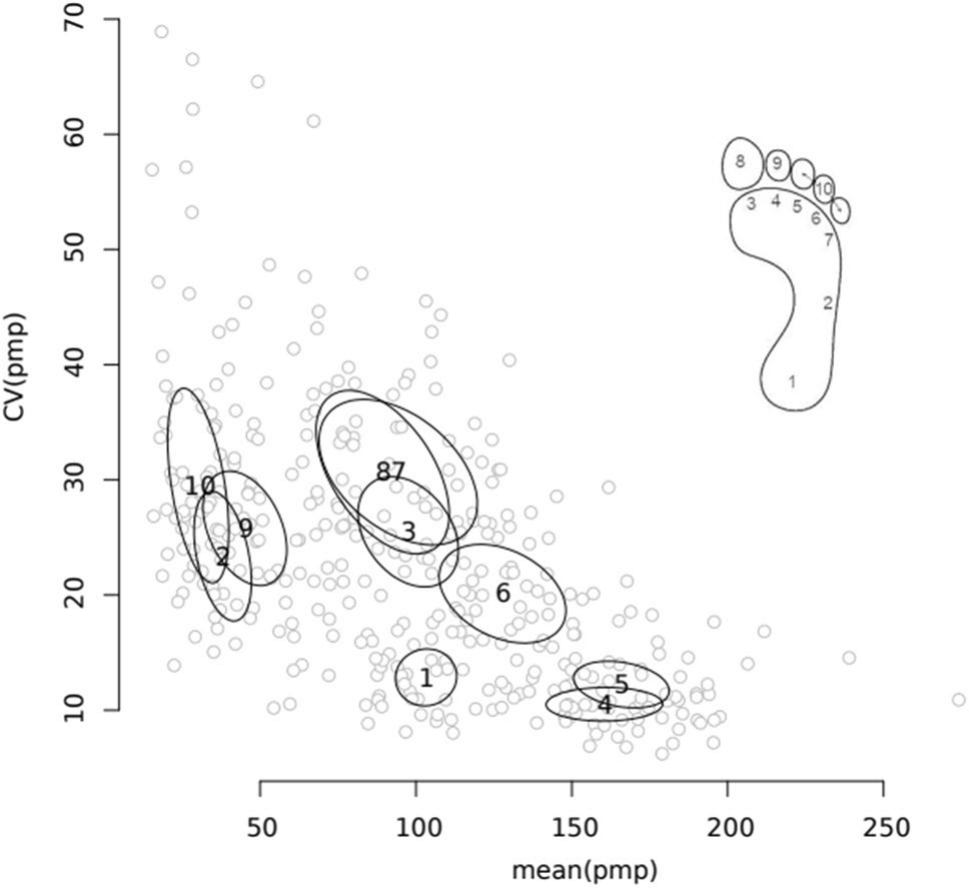
Intraindividual variation (CV) in relation to mean value of pmp index across foot areas (1: heel, 2: midfoot, 3–7: the five metatarsal heads, 8: hallux (first toe), 9: second toe and 10: third to fifth toes). Grey points symbolise the pairs of mean value and intraindividual CV for each mask of each participant. The centres of the ellipses describe pairs of the mean of both the average value (A) and the intra-individual variation (V^intra^) of the datasets for each mask, while the ellipses themselves describe the 1 SD distance from these centres.

We observed a clear individualisation of the pmp index, which affected all assessed foot areas to a generally similar extent, although there were noticeable differences between them. Each anatomical-functional area of the foot was characterised by the study-group-specific values for the mean and trial-by-trial variability of the pmp. We found that pmp index values vary both between masks and between individuals for each mask. The *p*-value of equality of the pmp mean values both across masks and across participants in each mask did not exceed 0.001. The estimated effect size (η^2^) of the differences between the means across masks is 1.89. The η^2^ of the differences between means across participants for each mask ranges from 0.50 to 1.82. The intra-individual means and intra-individual CVs of the pmp index for each mask can be seen in Table 1. The relationship between these quantities is illustrated in Fig. 2.

**Table 1:** Statistical description of pmp: group-wide average values of intra-individual means (A) and CVs (V^intra^) with related inter-individual dispersions (SD) and the η^2^ effect size of differences between means within participants, in mask-defined subsets.

Mask	A	SD(IM)	η^2^_participants_	V^intra^	SD(ICV)
1	103.22	14.62	0.78	13.39	3.68
2	37.99	13.72	1.23	23.95	8.40
3	97.46	24.02	0.50	25.93	7.13
4	160.42	27.85	1.82	10.7	2.17
5	165.85	22.88	0.98	12.28	3.01
6	127.74	30.33	0.82	20.34	6.39
7	94.27	37.87	0.90	30.57	9.42
8	89.30	31.87	0.65	32.4	10.58
9	45.10	20.01	1.00	27.21	7.40
10	30.10	14.60	1.07	32.15	12.61

**Figure 3:**
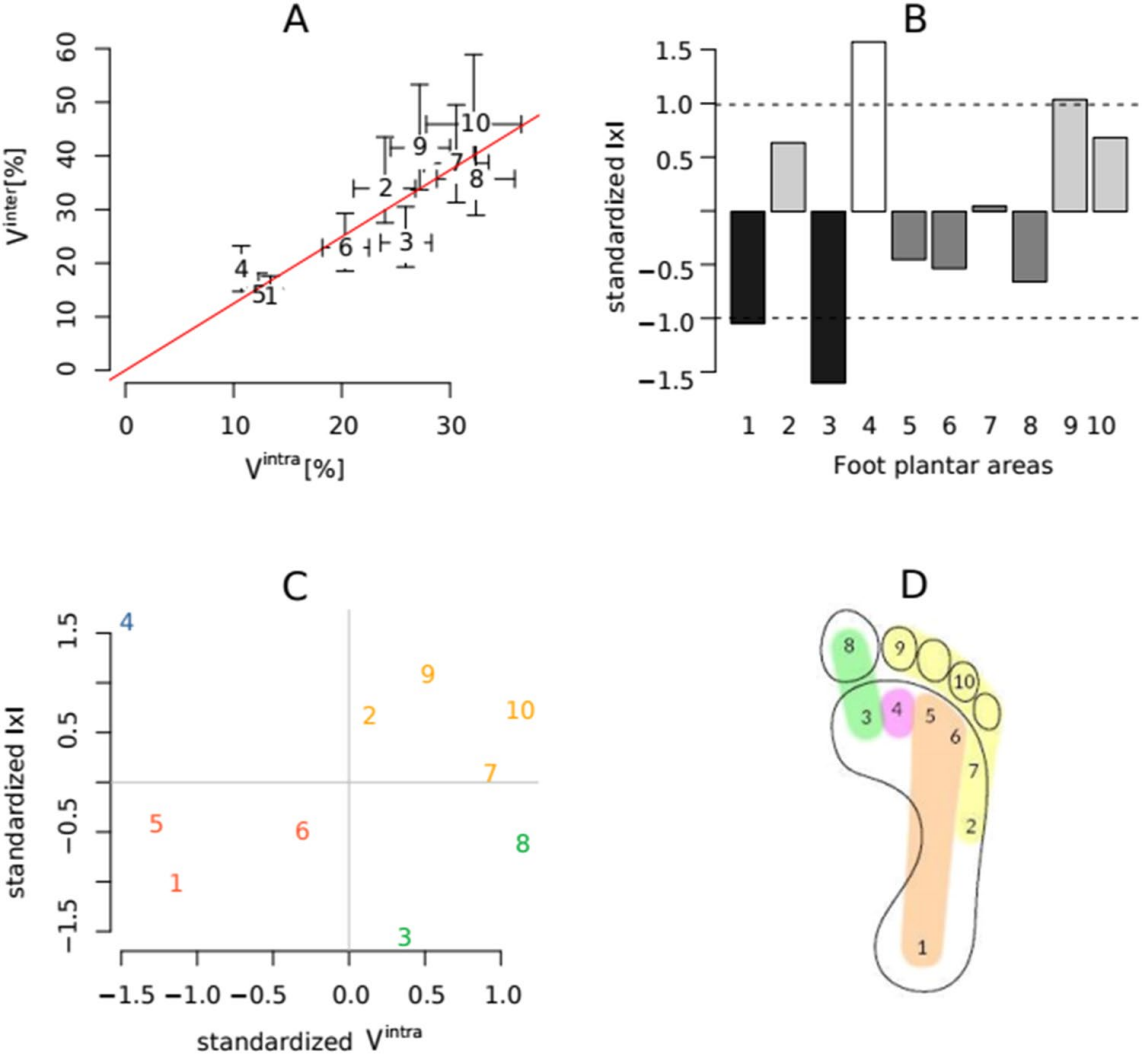
Biological variability in the contribution of particular foot parts to body weight shift during walking, assessed by analysis of pmp index values. (**A**) Highlighs a linear relationship (regression line: *V* = *mean(V*^*inter*^*/V*^*intra*^*)⋅V*^*intra*^) between the intra- and inter-individual variation as well as the diversity of their ratios between the masks independent of this relationship; the whiskers around each point represent 95% confidence intervals. (**B**) Illustrates the differences between the IxI for each mask and their mean value. The most obvious differences are between 1MTH (3) and 2MTH (4); the area of 1MTH (3) is the least, whereas 2MTH (4) is the most individualised. (**C**) The result of the k-means classification of these differences in terms of intra-individual CV, with the variation between 1MTH (3) and 2MTH (4) here reflecting the difference in their IxI values presented in (**B**). (**D**) Spatial illustration of the classification result, and shows the relationship of the variability and the individualised functioning of the different parts of the foot to their functional role in the body weight transfer process during walking. The Labelling of individual masks represents anatomical and functional foot plantar areas: 1: heel, 2: midfoot, 3–7: five metatarsal heads, 8: hallux (first toe), 9: second toe and 10: third to fifth toes.

The values of intra- and inter-individual variability (V^intra^ and V^inter^) and of the indices of individuality (IxI and IvI) for each mask are shown in Table 2. The V^intra^ and V^inter^ relate to each other in a linear manner (Fig. 3A), resulting in range of IxI values fairly narrow compared to their average. However, within this small range one can distinguish differences between these masks, especially between the lowest IxI for area 3, representing 1MTH, and the highest for area 4, representing 2MTH (Fig. 3B).

**Table 2:** Group-wide CVs ( V^intra^ and V^inter^) and indices of individuality (IxI and IvI) for each mask.

Mask	V^intra^	V^inter^	IxI	IvI
1	13.73 [11.09 16.49]	13.39 [12.13 14.65]	0.03 [− 0.09 0.12]	0.75 [0.60 0.93]
2	33.94 [29.56 39.93]	23.95 [21.12 26.79]	0.35 [0.34 0.40]	0.4 [0.25 0.55]
3	23.79 [19.62 28.37]	25.93 [23.58 28.28]	− 0.09 [− 0.18 0.00]	0.06 [− 0.07 0.18]
4	18.11 [12.95 24.61]	10.7 [9.99 11.41]	0.53 [0.26 0.77]	0.63 [0.49 0.78]
5	14.14 [10.27 18.19]	12.28 [11.32 13.25]	0.14 [− 0.10 0.32]	0.66 [0.53 0.78]
6	22.86 [19.32 27.52]	20.34 [18.22 22.45]	0.12 [0.06 0.20]	0.44 [0.31 0.57]
7	38.56 [30.65 47.18]	30.57 [27.56 33.59]	0.23 [0.11 0.34]	− 0.02 [− 0.13 0.09]
8	35.7 [28.88 43.76]	32.4 [28.80 36.00]	0.1 [0.00 0.20]	0.04 [− 0.06 0.18]
9	41.52 [34.77 48.72]	27.21 [24.45 29.97]	0.42 [0.35 0.49]	0.12 [− 0.19 0.36]
10	45.91 [38.37 57.5]	32.15 [27.79 36.51]	0.36 [0.32 0.45]	0.25 [0.21 0.35]

The k-means classification of pmp index IxI values in terms of intra-individual variability shown in Fig. 3C and illustrated in Fig. 3D clearly distinguishes some groups of plantar pressure areas. Within the areas analysed, mask 4 representing 2MTH stands out the most, with the highest IxI and the lowest variability. An inverse relationship of these classifiers characterises masks 3 and 8, representing the first ray of the foot. For masks 1, 5, 6, which cover areas corresponding to heel-forefoot line, IxI values are low at low variability. For masks 2, 7, 9, 10, all located around the rim of the foot, there is a high level of IxI at high level of variability.

**Figure 4:**
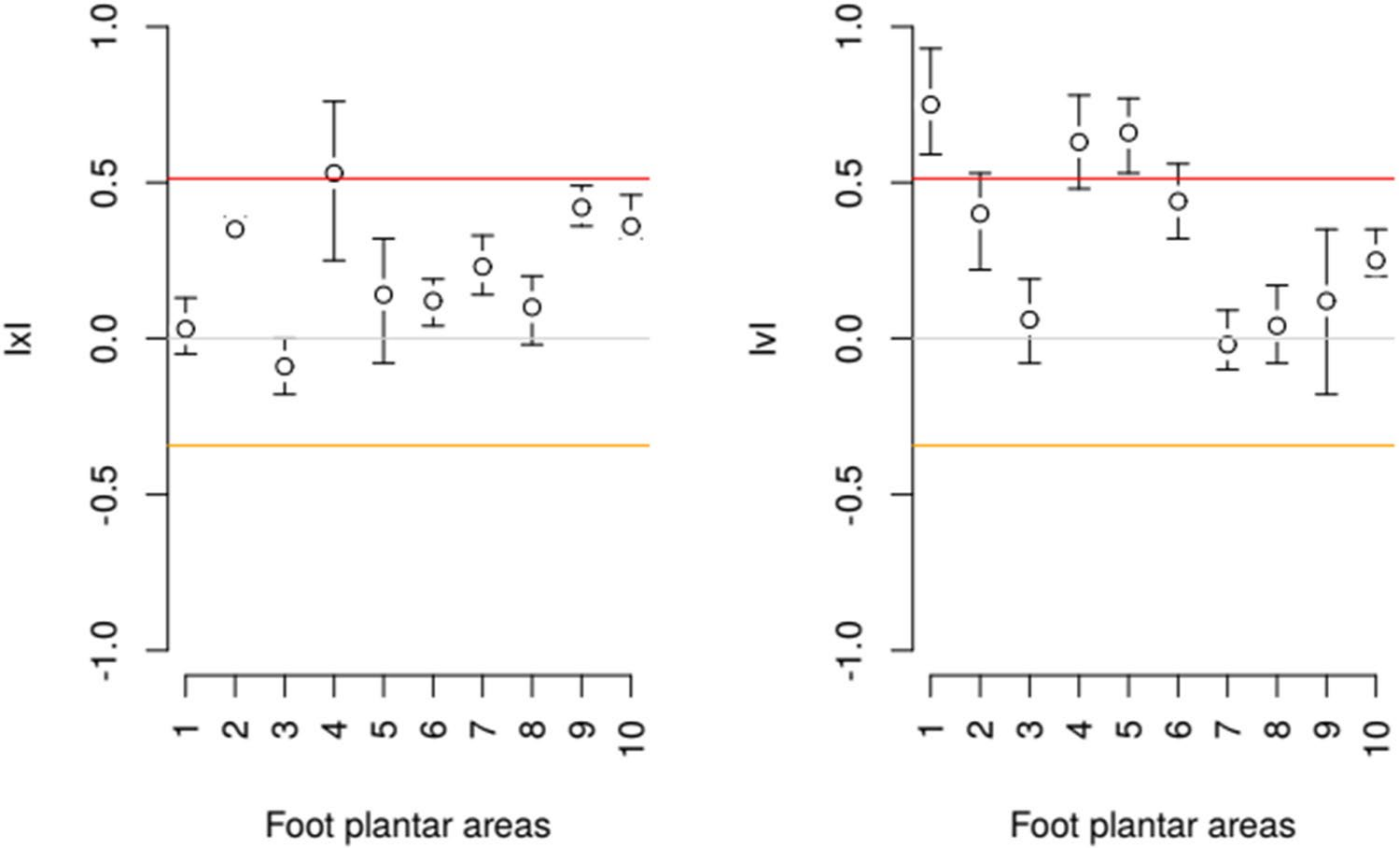
Individuality of the contribution of foot parts during body weight shifting, based on the pmp index. The red and yellow lines refer to thresholds that affect the decision of how to proceed with diagnostic assessment. None of the foot plantar areas has an individuality index that falls within the area perceived as strongly preferencing the use of a population reference interval. Foot plantar areas: 1—heel, 2—midfoot, 3–7—five metatarsal heads, 8—hallux (first toe), 9—second toe and 10—third to fifth toes.

None of the values of the IxI and IvI indices fell below − 0.336 (ln(1/1.4)), which is perceived as a decision threshold for the principle of setting a diagnostic reference based on population variation (Fig. 4). Furthermore, for almost all masks the indices of individuality exceeded 0 (V^intra^ > V^inter^). The sole exception was mask 3, corresponding to 1MTH, where the IxI values took on a value below 0, but still far from the recommended decision threshold for using a population-based reference standard. Overall, the indices of individuality took values closer to the range above 0.512 (logarithmic inverse of 0.6), which is perceived as strongly biased towards a diagnostic pattern based on internal rather than population variability.

## Discussion

This study, prompted by awareness of the individual variation within an overall population gait pattern and the complexity of foot functioning, expresses for the first time the need to consider biological variability in pedobarometric diagnosis. We investigated the relationship between intra- and inter-individual variability in the contribution of specific foot areas to weight shifting during walking in a homogeneous study group. Guided by the assumption that the functional space of each element of the kinematic chain corresponds to the role it plays in individual implementations of the movement task, we hypothesised that the individuality of functioning of the foot parts during walking would differ due to their differing roles in the weight shifting process. We found this differentiation in the values of the pedobarometric pmp index, which quantifies the contribution of different foot parts towards weight shifting. This biological variation described by an index of individuality corresponded to the role a foot part plays in weight-shifting, e.g., the first ray of the foot presented the lowest, and the second ray the highest level of the index of individuality. We concluded that because the space of formation of a unique gait pattern manifests in the regional differentiation of individuality of plantar pressures, the improvement of the quality of diagnostic assessment in pedobarometry requires the use of benchmark reference values unique to particular parts of the foot, and for enriching these benchmarks with indices of individuality expected from those foot parts.

The general pattern of weight shift during walking for the study group manifests itself by distinguishable ranges of levels of contribution for each foot area. In a homogeneous group of individuals, who share gender, age, body build, and lifestyle, the contribution levels clustered within particular ranges around each particular mean value for each individual participant, with individual trial-by-trial variability (Table 1). This means that an individual’s walking style uses only part of the overall available range of values that characterises the general model, which is demonstrated by the relative dominance of inter-individual variation compared to the intra-individual variation (V^inter^ over V^intra^). These two types of variation relate linearly to each other, so the ratios between them, expressed by indices of individuality, were of similar values across plantar surface areas (Fig. 3A), regardless of the level of their variability. This similarity of ratios evince yet again the existence of an overarching walking pattern. However, around this baseline, there are differences in the degree of individuality (Fig. 3B) that support the initial hypothesis. The result of the two-dimensional clustering of V^intra^ and IxI (Fig. 3C,D) further supports the hypothesis by revealing that the functional determination of the observed differences: the lowest individuality with high V^intra^ was observed in the first ray of the foot (1MTH and hallux), while the highest IxI with the lowest V^intra^ was observed in the second ray (2MTH and second toe).

As the characteristics of variability manifest the functional cause of the discovered regional differences in the indices of individuality, they extend the methodology of pedobarometry with the knowledge that the variation between individual gait patterns manifests to different degrees across foot parts. To the observation of an inverse correlation between mean regional plantar pressure and the coefficient of variation reported in other studies, we add the linear relationship between intra- and inter-individual coefficients of variation. By analysing the variation characteristics in the light of known theories of motor learning and motor control, we obtained an explanation of how motor control ensures the flexibility of task performance in the individual realisation of a common gait pattern by varying the intensity of individualisation and blurring it where necessary and possible. The first ray, which our findings showed is the least individualised, is the strongest part of the forefoot, and has a leading role in propulsion during the transfer of body weight to the other foot, and in adapting the course of this task to surrounding environmental conditions^37^. Its freedom of action, and consequently its variability, cannot, therefore, fall below the level necessary for flexible weight shifting—hence it was found to have much weaker DOF freezing. In contrast, the second ray and the mid-foot carry the restrictively optimised movement of the whole body, key for maintaining balance. The freedom of movement for these body parts is therefore more frozen, which in our study was reflected in higher index of individuality of their contributions in the motor task. It follows that in individual movement optimisation motor control varies the degree of DOF freezing across foot parts according to their role in the movement task, providing a level of variability necessary for the flexibility of whole foot function during cooperative performance of the weight shifting task. It seems that as individual characteristics mediate the functioning of each foot part in the movement task, motor control adapts the level of variability to the condition of the task performance. Thus, our study answers an important question in the field of motor control: namely, how motor control ensures individual consistency while maintaining the variability of performance necessary for adaptive flexibility.

Revealed regional differences in biological variation of foot part functioning explains why mechanical properties do not directly determine DOF freezing. For example, the intra-individual “trial-by-trial” variability of the first ray appears much higher than one could infer only from its load^31^. Even though the first ray carries most of the propulsion, it features high variability as it requires flexibility for this task^37^. We note that in terms of variability, the first metatarsal head (1MTH, i.e., mask 3) resembles toes more than the rest of the forefoot parts, although it transfers a significant part of the body weight. One should therefore see the variability of 1MTH work prevail over the variability of the second metatarsal head (2MTH, i.e., mask 4) more than would be explained by just the difference in load between them (ellipse figure). Further, although the first head (1MTH) has twice as much load as the fifth (5MTH), both act with similar variability. This deviation from the aforementioned inverse correlation between the mean value and coefficient of variation of the pressure exerted by the foot part on the ground, observed in this research, shows that the conditioning of the variability of the 1MTH movement results from less restrictive DOF freezing. It also demonstrates that pedobarometric data, in addition to providing information about biomechanical properties, also contains information about motor control-dependent functional characteristics of various foot parts. Thus, it further reinforces the need to consider biological variability as a manifestation of these characteristics while making diagnostic assessments.

Determining how to use biological variation in diagnostic assessment of weight shifting based on pedobarometric measurements requires consideration of two issues: how to implement assessment in relation to a reference standard, and what methods should be adopted to compare measurement data with the standard thus supplemented. It also requires consideration of the diversity of diagnostic evaluation objectives, and different conditions that might exist when making measurements for this evaluation. In the case of an assessment based on a single measurement, the information about the variability expressed by index of individuality and contained in the reference standard would help to estimate the strength of any deviation from the population mean value for a given group of patients. Variability information can also serve to estimate the value of the reference change value (RCV) index for assessing the effect of the change in weight-shifting over a certain time interval or between two states of patient functioning (e.g., preoperative and postoperative). As mentioned in “Methods” section, such an assessment requires the use of one of two types of variability index: subject or population, depending on the value of the individuality index. In our study group, the IxI values tended to approach the range perceived as strongly suggesting the need for the adoption of the intra-individual variability, rather than the range which would suggest using a population-based reference interval to estimate the RCV. For no area of the plantar surface of the foot did the IxI fall within the range that suggests the assessment against a population background. The values of the IvI index fall within a similar scope, applicable in an analogous analysis based on a series of measurements rather than a single measurement. This would allow the treatment of the variability estimated from the measurement session to be used as an additional evaluation index to the value itself (Fig. 4). To benefit from these opportunities, any diagnostic assessment should use a complex reference standard consisting of mean values and the intra-individual variabilities of the assessed indices together with their individuality coefficients. The differentiation of these parameters between different areas of the foot that we found means that the standard should have tailored sets of parameters for each of these areas. For the final establishment of a diagnostic framework, the development and validation of decision criteria based on comparisons with the reference standard and a selection of the most useful weight shift assessment indices for diagnostic assessment remains needed.

In order to present the ideas of diagnostic support for the reference information on variability, we have chosen to use the pmp index to describe plantar pressures. This index represents a normalised energy equivalent of the interaction between the parts of the foot and the ground, and we perceive it as valuable from a diagnostic assessment point of view. We formulated it as normalising the pressures exerted by each part of the foot during their individual contact with the ground by relating them to the average pressure exerted by the whole foot during the total contact time of the foot with the ground. Our measurement system uses artificial intelligence algorithms to extract the ground contact areas of each part of the foot. Through this method, and subsequent data normalisation, we reduced the effect on the analysed data of inter-individual anatomical and motor differences on the temporal sequence of the work of each part of the foot. A diagnostic benchmark extended by the set of regional values of the index of individuality, as characterising the inner functioning of the human foot during walking, gives the hope to improve the diagnostic value of pedobarometry.

## Conclusions

The degree of individualisation of the pressure values in different plantar areas corresponding to the parts of the foot involved in the force interaction with the ground during walking justifies the need to include indices of individuality of the descriptor of this pressure in the diagnostic benchmark forweight shift process. In this benchmark the index of individuality should describe each plantar area separately, as it shows considerable regional differences, particularly between the second ray with the highest value of this index and the first ray with the lowest value, that apparently reflects the relatively greater freedom of the bones of this ray to function during the shifting of the body weight while walking. We firmly believe that the presented conceptual approach that raises the necessity to consider biological variability in pedobarometric diagnosis has the potential to apply to any index of movement description that meets the requirements of standardisation.

## Data Availability

The datasets analysed during the current study available from the corresponding author on reasonable request.
